# Supplementary material for: Identification of photoperiod-induced specific miRNAs in the adrenal glands of Sunite sheep (Ovis aries)
Source: Front Vet Sci. 2022 Jul 22;9:888207. doi: 10.3389/fvets.2022.888207 (PMC9354845; doi:10.3389/fvets.2022.888207)
Supplement: Supplementary file 1 [file Data_Sheet_1.ZIP › Supplementary file/miRDeep2 file.zip!/miRDeep2 file/Novel_Structure/LP42A3/Novel_183.pdf]

Provisional ID	:	NC_019464.2_10062
Score total	:	120.5
Score for star read(s)	:	3.9
Score for read counts	:	112.8
Score for mfe	:	2.2
Score for randfold	:	1.6
Score for cons. seed	:	
Total read count	:	233
Mature read count	:	204
Loop read count	:	0
Star read count	:	29

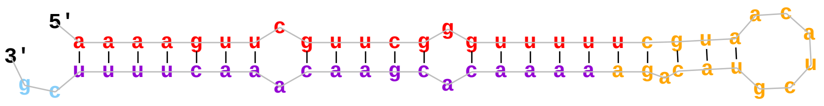

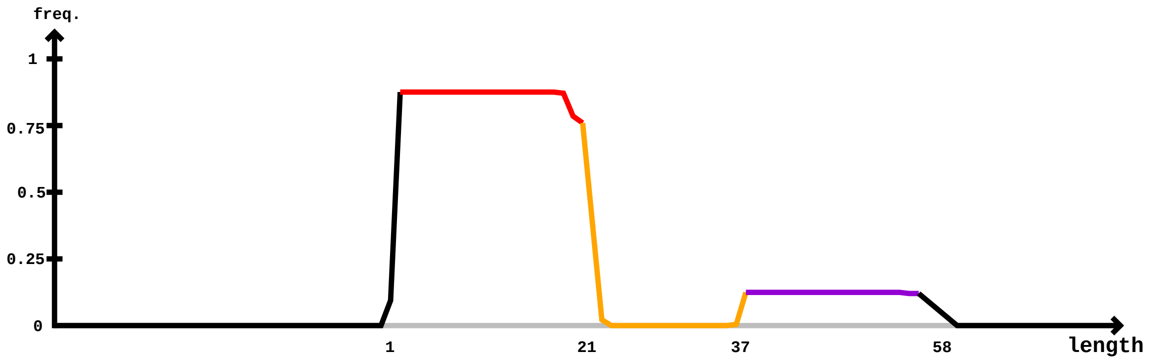

Mature **Star**

```
5'- guauucacaaaccauuaucauauuggguuggcugaaaaguucguucggguuuuucguaacaucguacagaaaaacacgaacaaacuuuucggccaacccaguauuucau   -3'    obs
    guauucacaaaccauuaucauauuggguuggcugaaaaguucguucggguuuuucguaacaucguacagaaaaacacgaacaaacuuuucggccaacccaguauuucau          exp
    ...................((((((((((((((((((((((.(((((.((((((((((......))).))))))).))))).))))))))))))))))))))))).....    reads    mm    sample
    .................................gaaaaguucguucggguu..........................................................    1        0     seq
    .................................gaaaaguucguucggguuu.........................................................    20       0     seq
    .................................Aaaaaguucguucggguuuu........................................................    1        1     seq
    ..................................aaaaguucguucggguuuu........................................................    5        0     seq
    ..................................aaaaguucguucggguuuuC.......................................................    10       1     seq
    ..................................aaaaguucguucggguuuuu.......................................................    99       0     seq
    ..................................aaaaguucguucUgguuuuu.......................................................    1        1     seq
    ..................................aaaaguucguucggguuuuuc......................................................    62       0     seq
    ..................................aaaaguucguucggguuuuucU.....................................................    3        1     seq
    ..................................aaaaguucguuUggguuuuucg.....................................................    2        1     seq
    .....................................................................aaaaacacgaacaaacuuuu....................    1        0     seq
    ......................................................................aaaacacgaacaaacuu......................    1        0     seq
    ......................................................................aaaacacgaacaaacuuuu....................    27       0     seq
```
